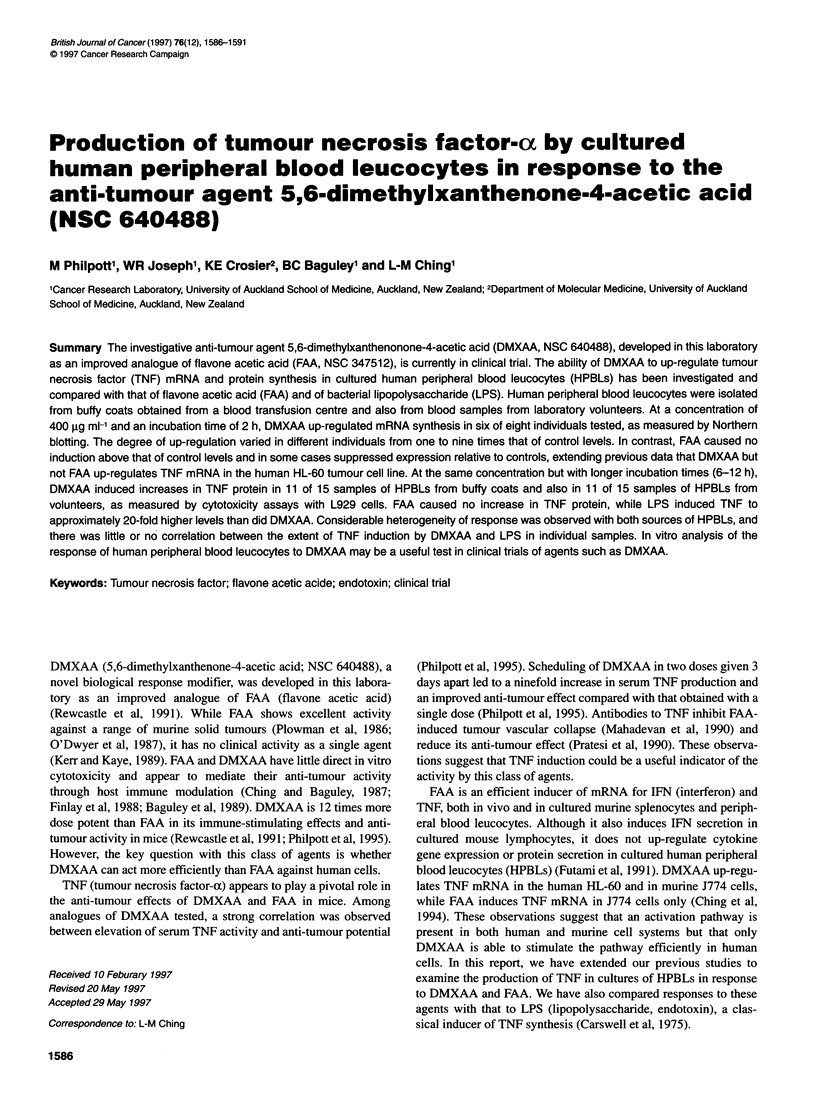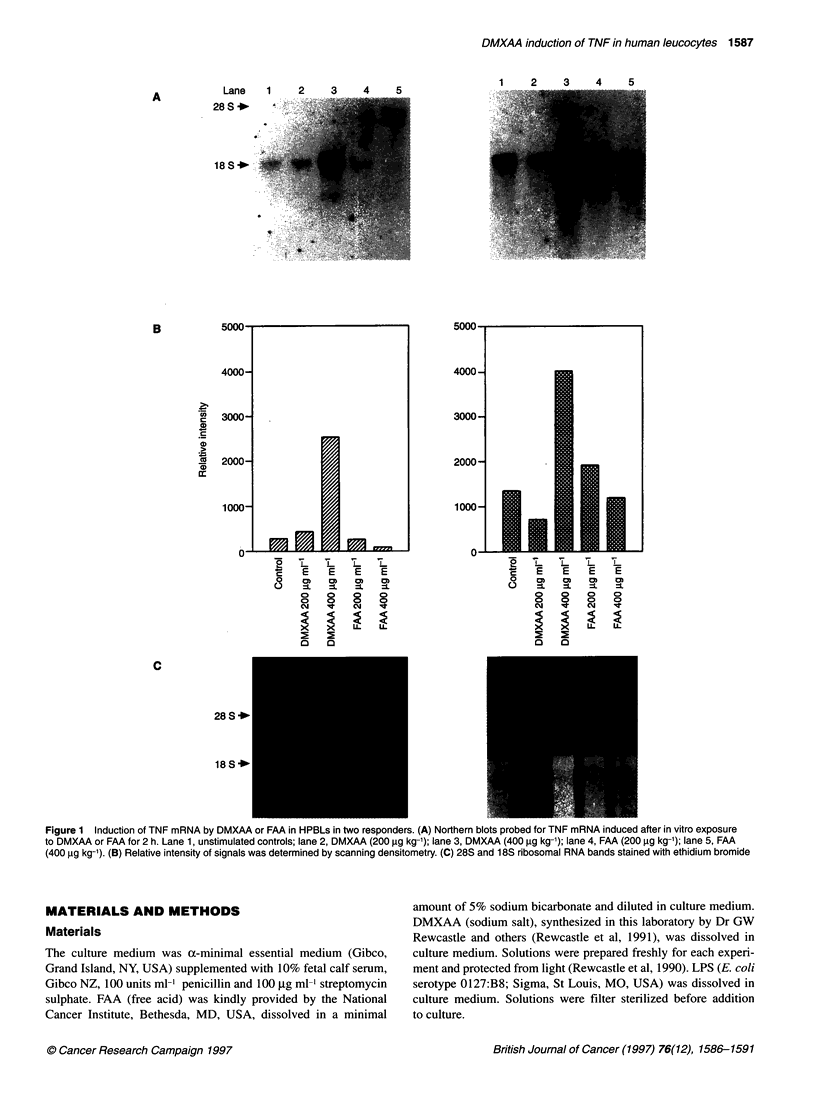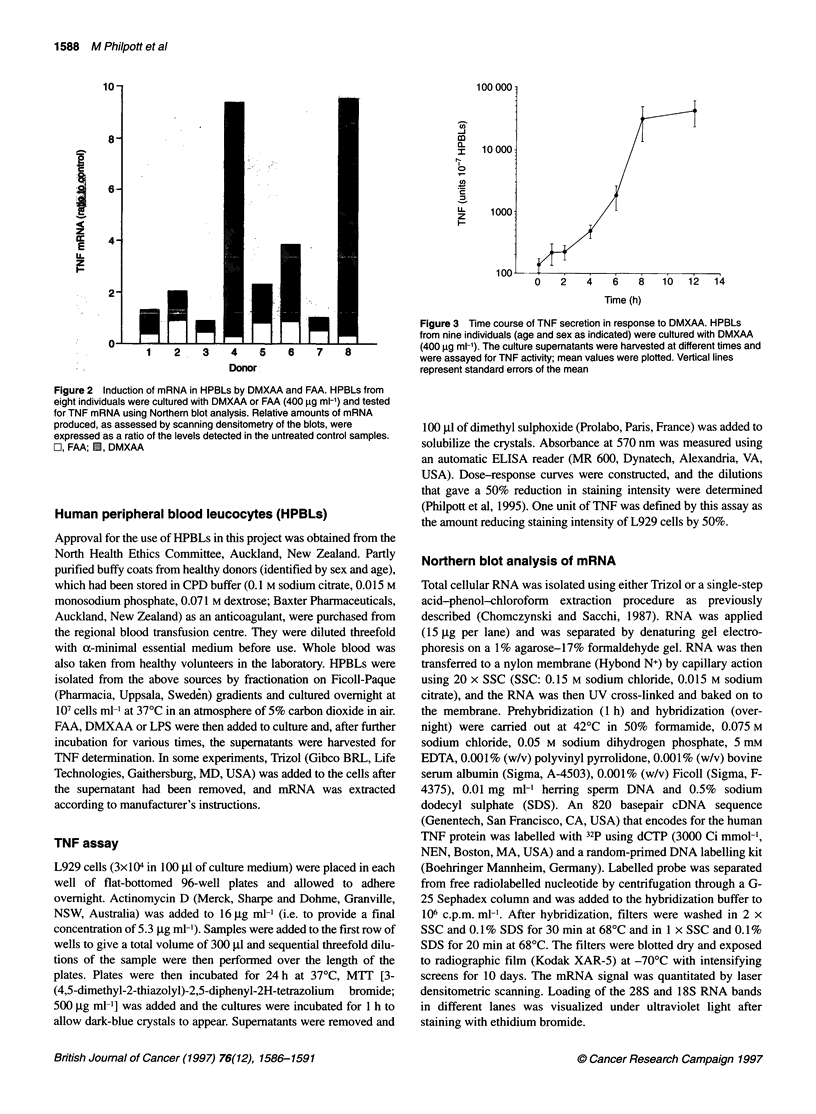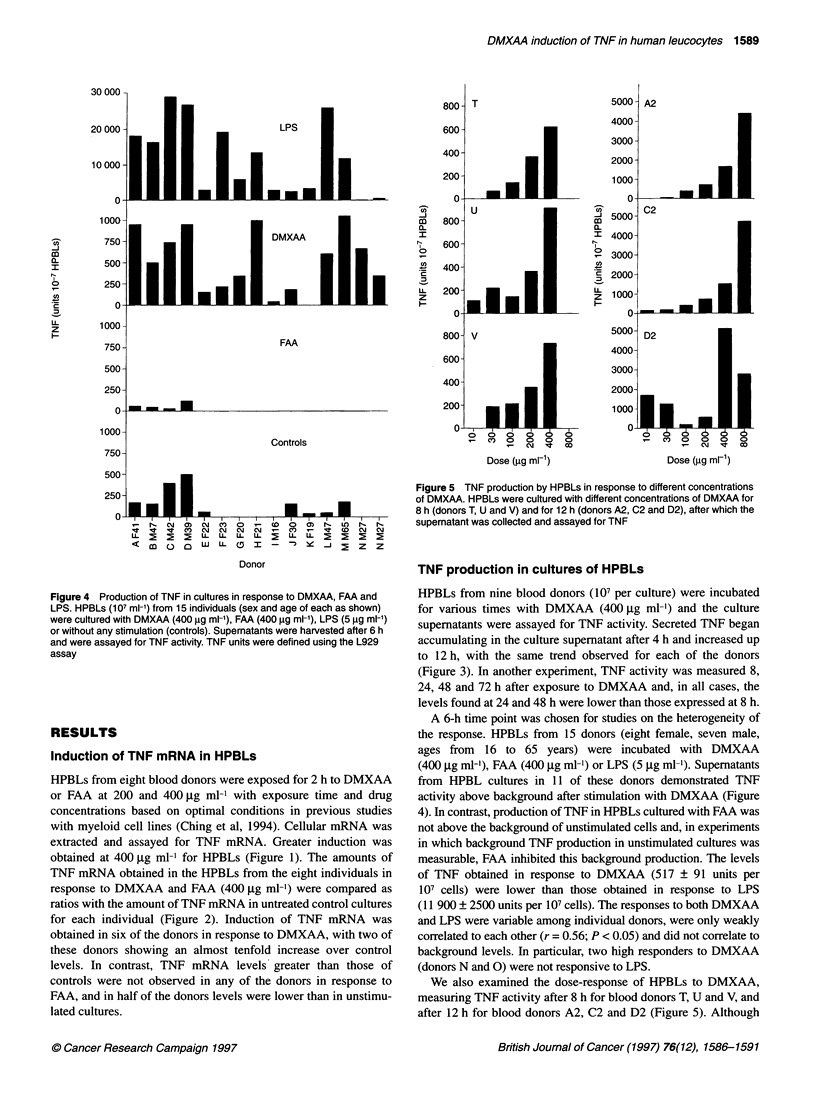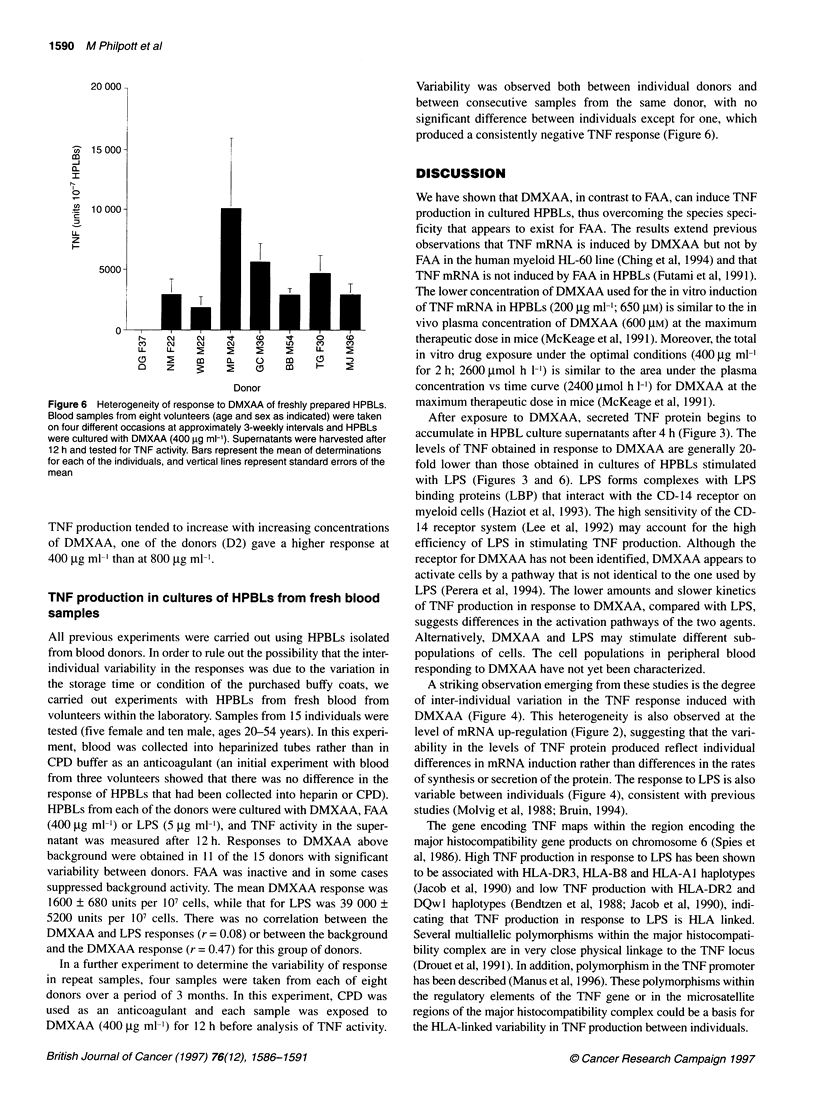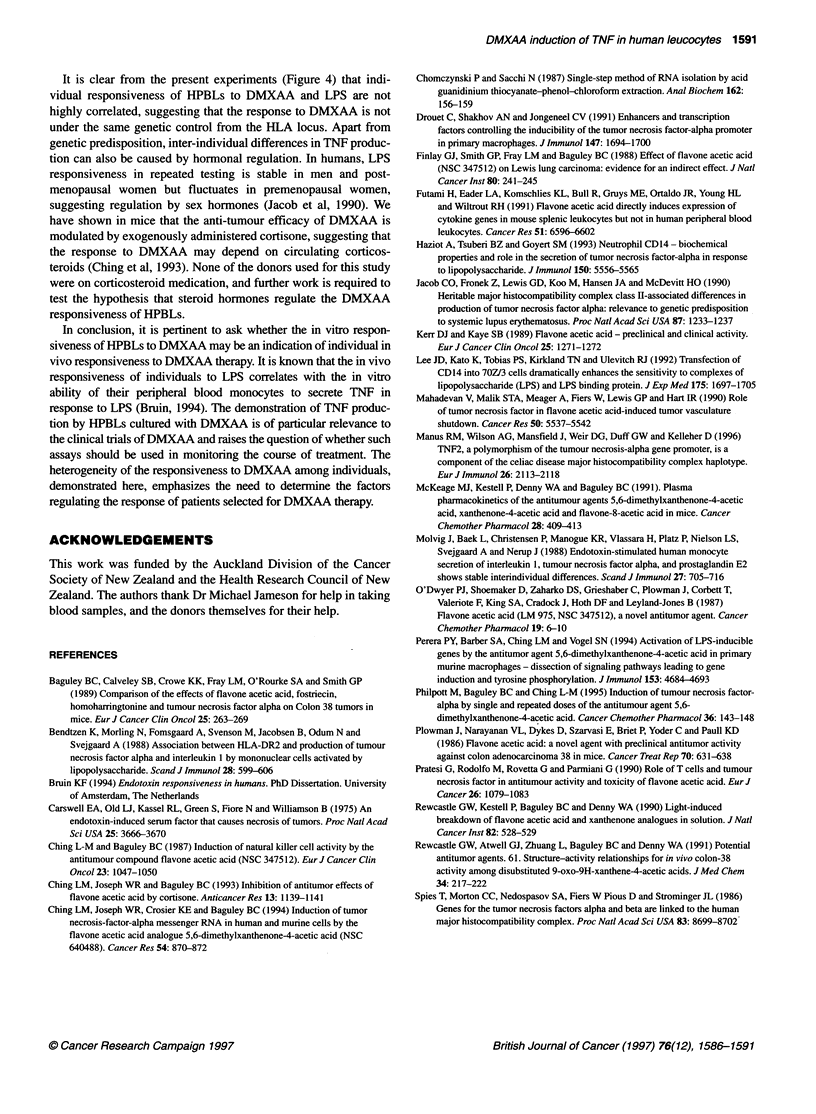# Production of tumour necrosis factor-alpha by cultured human peripheral blood leucocytes in response to the anti-tumour agent 5,6-dimethylxanthenone-4-acetic acid (NSC 640488).

**DOI:** 10.1038/bjc.1997.601

**Published:** 1997

**Authors:** M. Philpott, W. R. Joseph, K. E. Crosier, B. C. Baguley, L. M. Ching

**Affiliations:** Cancer Research Laboratory, University of Auckland School of Medicine, New Zealand.

## Abstract

**Images:**


British Journal of Cancer (1997) 76(12), 1586–1591
© 1997 Cancer Research Campaign

### Production of tumour necrosis factor-α by cultured human peripheral blood leucocytes in response to the anti-tumour agent 5,6-dimethylxanthenone-4-acetic acid (NSC 640488)

**M Philpott[1], WR Joseph[1], KE Crosier[2], BC Baguley[1] and L-M Ching[1]**

[1]Cancer Research Laboratory, University of Auckland School of Medicine, Auckland, New Zealand; [2]Department of Molecular Medicine, University of Auckland School of Medicine, Auckland, New Zealand

**Summary** The investigative anti-tumour agent 5,6-dimethylxanthenononе-4-acetic acid (DMXAA, NSC 640488), developed in this laboratory as an improved analogue of flavone acetic acid (FAA, NSC 347512), is currently in clinical trial. The ability of DMXAA to up-regulate tumour necrosis factor (TNF) mRNA and protein synthesis in cultured human peripheral blood leucocytes (HPBLs) has been investigated and compared with that of flavone acetic acid (FAA) and of bacterial lipopolysaccharide (LPS). Human peripheral blood leucocytes were isolated from buffy coats obtained from a blood transfusion centre and also from blood samples from laboratory volunteers. At a concentration of 400 μg $ml^{-1}$ and an incubation time of 2 h, DMXAA up-regulated mRNA synthesis in six of eight individuals tested, as measured by Northern blotting. The degree of up-regulation varied in different individuals from one to nine times that of control levels. In contrast, FAA caused no induction above that of control levels and in some cases suppressed expression relative to controls, extending previous data that DMXAA but not FAA up-regulates TNF mRNA in the human HL-60 tumour cell line. At the same concentration but with longer incubation times (6–12 h), DMXAA induced increases in TNF protein in 11 of 15 samples of HPBLs from buffy coats and also in 11 of 15 samples of HPBLs from volunteers, as measured by cytotoxicity assays with L929 cells. FAA caused no increase in TNF protein, while LPS induced TNF to approximately 20-fold higher levels than did DMXAA. Considerable heterogeneity of response was observed with both sources of HPBLs, and there was little or no correlation between the extent of TNF induction by DMXAA and LPS in individual samples. In vitro analysis of the response of human peripheral blood leucocytes to DMXAA may be a useful test in clinical trials of agents such as DMXAA.

**Keywords:** Tumour necrosis factor; flavone acetic acide; endotoxin; clinical trial

DMXAA (5,6-dimethylxanthenone-4-acetic acid; NSC 640488), a novel biological response modifier, was developed in this laboratory as an improved analogue of FAA (flavone acetic acid) (Rewcastle et al, 1991). While FAA shows excellent activity against a range of murine solid tumours (Plowman et al, 1986; O'Dwyer et al, 1987), it has no clinical activity as a single agent (Kerr and Kaye, 1989). FAA and DMXAA have little direct in vitro cytotoxicity and appear to mediate their anti-tumour activity through host immune modulation (Ching and Baguley, 1987; Finlay et al, 1988; Baguley et al, 1989). DMXAA is 12 times more dose potent than FAA in its immune-stimulating effects and anti-tumour activity in mice (Rewcastle et al, 1991; Philpott et al, 1995). However, the key question with this class of agents is whether DMXAA can act more efficiently than FAA against human cells.

TNF (tumour necrosis factor-α) appears to play a pivotal role in the anti-tumour effects of DMXAA and FAA in mice. Among analogues of DMXAA tested, a strong correlation was observed between elevation of serum TNF activity and anti-tumour potential (Philpott et al, 1995). Scheduling of DMXAA in two doses given 3 days apart led to a ninefold increase in serum TNF production and an improved anti-tumour effect compared with that obtained with a single dose (Philpott et al, 1995). Antibodies to TNF inhibit FAA-induced tumour vascular collapse (Mahadevan et al, 1990) and reduce its anti-tumour effect (Pratesi et al, 1990). These observations suggest that TNF induction could be a useful indicator of the activity by this class of agents.

FAA is an efficient inducer of mRNA for IFN (interferon) and TNF, both in vivo and in cultured murine splenocytes and peripheral blood leucocytes. Although it also induces IFN secretion in cultured mouse lymphocytes, it does not up-regulate cytokine gene expression or protein secretion in cultured human peripheral blood leucocytes (HPBLs) (Futami et al, 1991). DMXAA up-regulates TNF mRNA in the human HL-60 and in murine J774 cells, while FAA induces TNF mRNA in J774 cells only (Ching et al, 1994). These observations suggest that an activation pathway is present in both human and murine cell systems but that only DMXAA is able to stimulate the pathway efficiently in human cells. In this report, we have extended our previous studies to examine the production of TNF in cultures of HPBLs in response to DMXAA and FAA. We have also compared responses to these agents with that to LPS (lipopolysaccharide, endotoxin), a classical inducer of TNF synthesis (Carswell et al, 1975).

*Received 10 Feburary 1997*
*Revised 20 May 1997*
*Accepted 29 May 1997*

*Correspondence to:* L-M Ching

**Figure 1** Induction of TNF mRNA by DMXAA or FAA in HPBLs in two responders. (**A**) Northern blots probed for TNF mRNA induced after in vitro exposure to DMXAA or FAA for 2 h. Lane 1, unstimulated controls; lane 2, DMXAA (200 µg $kg^{-1}$); lane 3, DMXAA (400 µg $kg^{-1}$); lane 4, FAA (200 µg $kg^{-1}$); lane 5, FAA (400 µg $kg^{-1}$). (**B**) Relative intensity of signals was determined by scanning densitometry. (**C**) 28S and 18S ribosomal RNA bands stained with ethidium bromide

### MATERIALS AND METHODS

### Materials

The culture medium was α-minimal essential medium (Gibco, Grand Island, NY, USA) supplemented with 10% fetal calf serum, Gibco NZ, 100 units $ml^{-1}$ penicillin and 100 µg $ml^{-1}$ streptomycin sulphate. FAA (free acid) was kindly provided by the National Cancer Institute, Bethesda, MD, USA, dissolved in a minimal amount of 5% sodium bicarbonate and diluted in culture medium. DMXAA (sodium salt), synthesized in this laboratory by Dr GW Rewcastle and others (Rewcastle et al, 1991), was dissolved in culture medium. Solutions were prepared freshly for each experiment and protected from light (Rewcastle et al, 1990). LPS (*E. coli* serotype 0127:B8; Sigma, St Louis, MO, USA) was dissolved in culture medium. Solutions were filter sterilized before addition to culture.

© *Cancer Research Campaign 1997* 

**Figure 2** Induction of mRNA in HPBLs by DMXAA and FAA. HPBLs from eight individuals were cultured with DMXAA or FAA (400 μg ml$^{-1}$) and tested for TNF mRNA using Northern blot analysis. Relative amounts of mRNA produced, as assessed by scanning densitometry of the blots, were expressed as a ratio of the levels detected in the untreated control samples. □, FAA; ▨, DMXAA

**Figure 3** Time course of TNF secretion in response to DMXAA. HPBLs from nine individuals (age and sex as indicated) were cultured with DMXAA (400 μg ml$^{-1}$). The culture supernatants were harvested at different times and were assayed for TNF activity; mean values were plotted. Vertical lines represent standard errors of the mean

### Human peripheral blood leucocytes (HPBLs)

Approval for the use of HPBLs in this project was obtained from the North Health Ethics Committee, Auckland, New Zealand. Partly purified buffy coats from healthy donors (identified by sex and age), which had been stored in CPD buffer (0.1 M sodium citrate, 0.015 M monosodium phosphate, 0.071 M dextrose; Baxter Pharmaceuticals, Auckland, New Zealand) as an anticoagulant, were purchased from the regional blood transfusion centre. They were diluted threefold with α-minimal essential medium before use. Whole blood was also taken from healthy volunteers in the laboratory. HPBLs were isolated from the above sources by fractionation on Ficoll-Paque (Pharmacia, Uppsala, Sweden) gradients and cultured overnight at $10^7$ cells ml$^{-1}$ at 37°C in an atmosphere of 5% carbon dioxide in air. FAA, DMXAA or LPS were then added to culture and, after further incubation for various times, the supernatants were harvested for TNF determination. In some experiments, Trizol (Gibco BRL, Life Technologies, Gaithersburg, MD, USA) was added to the cells after the supernatant had been removed, and mRNA was extracted according to manufacturer's instructions.

### TNF assay

L929 cells ($3\times10^4$ in 100 μl of culture medium) were placed in each well of flat-bottomed 96-well plates and allowed to adhere overnight. Actinomycin D (Merck, Sharpe and Dohme, Granville, NSW, Australia) was added to 16 μg ml$^{-1}$ (i.e. to provide a final concentration of 5.3 μg ml$^{-1}$). Samples were added to the first row of wells to give a total volume of 300 μl and sequential threefold dilutions of the sample were then performed over the length of the plates. Plates were then incubated for 24 h at 37°C, MTT [3-(4,5-dimethyl-2-thiazolyl)-2,5-diphenyl-2H-tetrazolium bromide; 500 μg ml$^{-1}$] was added and the cultures were incubated for 1 h to allow dark-blue crystals to appear. Supernatants were removed and 100 μl of dimethyl sulphoxide (Prolabo, Paris, France) was added to solubilize the crystals. Absorbance at 570 nm was measured using an automatic ELISA reader (MR 600, Dynatech, Alexandria, VA, USA). Dose–response curves were constructed, and the dilutions that gave a 50% reduction in staining intensity were determined (Philpott et al, 1995). One unit of TNF was defined by this assay as the amount reducing staining intensity of L929 cells by 50%.

### Northern blot analysis of mRNA

Total cellular RNA was isolated using either Trizol or a single-step acid–phenol–chloroform extraction procedure as previously described (Chomczynski and Sacchi, 1987). RNA was applied (15 μg per lane) and was separated by denaturing gel electrophoresis on a 1% agarose–17% formaldehyde gel. RNA was then transferred to a nylon membrane (Hybond N+) by capillary action using 20 × SSC (SSC: 0.15 M sodium chloride, 0.015 M sodium citrate), and the RNA was then UV cross-linked and baked on to the membrane. Prehybridization (1 h) and hybridization (overnight) were carried out at 42°C in 50% formamide, 0.075 M sodium chloride, 0.05 M sodium dihydrogen phosphate, 5 mM EDTA, 0.001% (w/v) polyvinyl pyrrolidone, 0.001% (w/v) bovine serum albumin (Sigma, A-4503), 0.001% (w/v) Ficoll (Sigma, F-4375), 0.01 mg ml$^{-1}$ herring sperm DNA and 0.5% sodium dodecyl sulphate (SDS). An 820 basepair cDNA sequence (Genentech, San Francisco, CA, USA) that encodes for the human TNF protein was labelled with $^{32}P$ using dCTP (3000 Ci mmol$^{-1}$, NEN, Boston, MA, USA) and a random-primed DNA labelling kit (Boehringer Mannheim, Germany). Labelled probe was separated from free radiolabelled nucleotide by centrifugation through a G-25 Sephadex column and was added to the hybridization buffer to $10^6$ c.p.m. ml$^{-1}$. After hybridization, filters were washed in 2 × SSC and 0.1% SDS for 30 min at 68°C and in 1 × SSC and 0.1% SDS for 20 min at 68°C. The filters were blotted dry and exposed to radiographic film (Kodak XAR-5) at –70°C with intensifying screens for 10 days. The mRNA signal was quantitated by laser densitometric scanning. Loading of the 28S and 18S RNA bands in different lanes was visualized under ultraviolet light after staining with ethidium bromide.

© Cancer Research Campaign 1997

**Figure 4** Production of TNF in cultures in response to DMXAA, FAA and LPS. HPBLs ($10^7$ ml$^{-1}$) from 15 individuals (sex and age of each as shown) were cultured with DMXAA (400 µg ml$^{-1}$), FAA (400 µg ml$^{-1}$), LPS (5 µg ml$^{-1}$) or without any stimulation (controls). Supernatants were harvested after 6 h and were assayed for TNF activity. TNF units were defined using the L929 assay

**Figure 5** TNF production by HPBLs in response to different concentrations of DMXAA. HPBLs were cultured with different concentrations of DMXAA for 8 h (donors T, U and V) and for 12 h (donors A2, C2 and D2), after which the supernatant was collected and assayed for TNF

### RESULTS

### Induction of TNF mRNA in HPBLs

HPBLs from eight blood donors were exposed for 2 h to DMXAA or FAA at 200 and 400 µg ml$^{-1}$ with exposure time and drug concentrations based on optimal conditions in previous studies with myeloid cell lines (Ching et al, 1994). Cellular mRNA was extracted and assayed for TNF mRNA. Greater induction was obtained at 400 µg ml$^{-1}$ for HPBLs (Figure 1). The amounts of TNF mRNA obtained in the HPBLs from the eight individuals in response to DMXAA and FAA (400 µg ml$^{-1}$) were compared as ratios with the amount of TNF mRNA in untreated control cultures for each individual (Figure 2). Induction of TNF mRNA was obtained in six of the donors in response to DMXAA, with two of these donors showing an almost tenfold increase over control levels. In contrast, TNF mRNA levels greater than those of controls were not observed in any of the donors in response to FAA, and in half of the donors levels were lower than in unstimulated cultures.

### TNF production in cultures of HPBLs

HPBLs from nine blood donors ($10^7$ per culture) were incubated for various times with DMXAA (400 µg ml$^{-1}$) and the culture supernatants were assayed for TNF activity. Secreted TNF began accumulating in the culture supernatant after 4 h and increased up to 12 h, with the same trend observed for each of the donors (Figure 3). In another experiment, TNF activity was measured 8, 24, 48 and 72 h after exposure to DMXAA and, in all cases, the levels found at 24 and 48 h were lower than those expressed at 8 h.

A 6-h time point was chosen for studies on the heterogeneity of the response. HPBLs from 15 donors (eight female, seven male, ages from 16 to 65 years) were incubated with DMXAA (400 µg ml$^{-1}$), FAA (400 µg ml$^{-1}$) or LPS (5 µg ml$^{-1}$). Supernatants from HPBL cultures in 11 of these donors demonstrated TNF activity above background after stimulation with DMXAA (Figure 4). In contrast, production of TNF in HPBLs cultured with FAA was not above the background of unstimulated cells and, in experiments in which background TNF production in unstimulated cultures was measurable, FAA inhibited this background production. The levels of TNF obtained in response to DMXAA (517 ± 91 units per $10^7$ cells) were lower than those obtained in response to LPS (11 900 ± 2500 units per $10^7$ cells). The responses to both DMXAA and LPS were variable among individual donors, were only weakly correlated to each other ($r = 0.56$; $P < 0.05$) and did not correlate to background levels. In particular, two high responders to DMXAA (donors N and O) were not responsive to LPS.

We also examined the dose-response of HPBLs to DMXAA, measuring TNF activity after 8 h for blood donors T, U and V, and after 12 h for blood donors A2, C2 and D2 (Figure 5). Although

© Cancer Research Campaign 1997

**Figure 6** Heterogeneity of response to DMXAA of freshly prepared HPBLs. Blood samples from eight volunteers (age and sex as indicated) were taken on four different occasions at approximately 3-weekly intervals and HPBLs were cultured with DMXAA (400 μg $ml^{-1}$). Supernatants were harvested after 12 h and tested for TNF activity. Bars represent the mean of determinations for each of the individuals, and vertical lines represent standard errors of the mean

TNF production tended to increase with increasing concentrations of DMXAA, one of the donors (D2) gave a higher response at 400 μg $ml^{-1}$ than at 800 μg $ml^{-1}$.

### TNF production in cultures of HPBLs from fresh blood samples

All previous experiments were carried out using HPBLs isolated from blood donors. In order to rule out the possibility that the inter-individual variability in the responses was due to the variation in the storage time or condition of the purchased buffy coats, we carried out experiments with HPBLs from fresh blood from volunteers within the laboratory. Samples from 15 individuals were tested (five female and ten male, ages 20–54 years). In this experiment, blood was collected into heparinized tubes rather than in CPD buffer as an anticoagulant (an initial experiment with blood from three volunteers showed that there was no difference in the response of HPBLs that had been collected into heparin or CPD). HPBLs from each of the donors were cultured with DMXAA, FAA (400 μg $ml^{-1}$) or LPS (5 μg $ml^{-1}$), and TNF activity in the supernatant was measured after 12 h. Responses to DMXAA above background were obtained in 11 of the 15 donors with significant variability between donors. FAA was inactive and in some cases suppressed background activity. The mean DMXAA response was 1600 ± 680 units per $10^7$ cells, while that for LPS was 39 000 ± 5200 units per $10^7$ cells. There was no correlation between the DMXAA and LPS responses ($r = 0.08$) or between the background and the DMXAA response ($r = 0.47$) for this group of donors.

In a further experiment to determine the variability of response in repeat samples, four samples were taken from each of eight donors over a period of 3 months. In this experiment, CPD was used as an anticoagulant and each sample was exposed to DMXAA (400 μg $ml^{-1}$) for 12 h before analysis of TNF activity. Variability was observed both between individual donors and between consecutive samples from the same donor, with no significant difference between individuals except for one, which produced a consistently negative TNF response (Figure 6).

### DISCUSSION

We have shown that DMXAA, in contrast to FAA, can induce TNF production in cultured HPBLs, thus overcoming the species specificity that appears to exist for FAA. The results extend previous observations that TNF mRNA is induced by DMXAA but not by FAA in the human myeloid HL-60 line (Ching et al, 1994) and that TNF mRNA is not induced by FAA in HPBLs (Futami et al, 1991). The lower concentration of DMXAA used for the in vitro induction of TNF mRNA in HPBLs (200 μg $ml^{-1}$; 650 μM) is similar to the in vivo plasma concentration of DMXAA (600 μM) at the maximum therapeutic dose in mice (McKeage et al, 1991). Moreover, the total in vitro drug exposure under the optimal conditions (400 μg $ml^{-1}$ for 2 h; 2600 μmol h $l^{-1}$) is similar to the area under the plasma concentration vs time curve (2400 μmol h $l^{-1}$) for DMXAA at the maximum therapeutic dose in mice (McKeage et al, 1991).

After exposure to DMXAA, secreted TNF protein begins to accumulate in HPBL culture supernatants after 4 h (Figure 3). The levels of TNF obtained in response to DMXAA are generally 20-fold lower than those obtained in cultures of HPBLs stimulated with LPS (Figures 3 and 6). LPS forms complexes with LPS binding proteins (LBP) that interact with the CD-14 receptor on myeloid cells (Haziot et al, 1993). The high sensitivity of the CD-14 receptor system (Lee et al, 1992) may account for the high efficiency of LPS in stimulating TNF production. Although the receptor for DMXAA has not been identified, DMXAA appears to activate cells by a pathway that is not identical to the one used by LPS (Perera et al, 1994). The lower amounts and slower kinetics of TNF production in response to DMXAA, compared with LPS, suggests differences in the activation pathways of the two agents. Alternatively, DMXAA and LPS may stimulate different sub-populations of cells. The cell populations in peripheral blood responding to DMXAA have not yet been characterized.

A striking observation emerging from these studies is the degree of inter-individual variation in the TNF response induced with DMXAA (Figure 4). This heterogeneity is also observed at the level of mRNA up-regulation (Figure 2), suggesting that the variability in the levels of TNF protein produced reflect individual differences in mRNA induction rather than differences in the rates of synthesis or secretion of the protein. The response to LPS is also variable between individuals (Figure 4), consistent with previous studies (Molvig et al, 1988; Bruin, 1994).

The gene encoding TNF maps within the region encoding the major histocompatibility gene products on chromosome 6 (Spies et al, 1986). High TNF production in response to LPS has been shown to be associated with HLA-DR3, HLA-B8 and HLA-A1 haplotypes (Jacob et al, 1990) and low TNF production with HLA-DR2 and DQw1 haplotypes (Bendtzen et al, 1988; Jacob et al, 1990), indicating that TNF production in response to LPS is HLA linked. Several multiallelic polymorphisms within the major histocompatibility complex are in very close physical linkage to the TNF locus (Drouet et al, 1991). In addition, polymorphism in the TNF promoter has been described (Manus et al, 1996). These polymorphisms within the regulatory elements of the TNF gene or in the microsatellite regions of the major histocompatibility complex could be a basis for the HLA-linked variability in TNF production between individuals.

© Cancer Research Campaign 1997

It is clear from the present experiments (Figure 4) that individual responsiveness of HPBLs to DMXAA and LPS are not highly correlated, suggesting that the response to DMXAA is not under the same genetic control from the HLA locus. Apart from genetic predisposition, inter-individual differences in TNF production can also be caused by hormonal regulation. In humans, LPS responsiveness in repeated testing is stable in men and post-menopausal women but fluctuates in premenopausal women, suggesting regulation by sex hormones (Jacob et al, 1990). We have shown in mice that the anti-tumour efficacy of DMXAA is modulated by exogenously administered cortisone, suggesting that the response to DMXAA may depend on circulating corticosteroids (Ching et al, 1993). None of the donors used for this study were on corticosteroid medication, and further work is required to test the hypothesis that steroid hormones regulate the DMXAA responsiveness of HPBLs.

In conclusion, it is pertinent to ask whether the in vitro responsiveness of HPBLs to DMXAA may be an indication of individual in vivo responsiveness to DMXAA therapy. It is known that the in vivo responsiveness of individuals to LPS correlates with the in vitro ability of their peripheral blood monocytes to secrete TNF in response to LPS (Bruin, 1994). The demonstration of TNF production by HPBLs cultured with DMXAA is of particular relevance to the clinical trials of DMXAA and raises the question of whether such assays should be used in monitoring the course of treatment. The heterogeneity of the responsiveness to DMXAA among individuals, demonstrated here, emphasizes the need to determine the factors regulating the response of patients selected for DMXAA therapy.

### ACKNOWLEDGEMENTS

This work was funded by the Auckland Division of the Cancer Society of New Zealand and the Health Research Council of New Zealand. The authors thank Dr Michael Jameson for help in taking blood samples, and the donors themselves for their help.

### REFERENCES

Baguley BC, Calveley SB, Crowe KK, Fray LM, O'Rourke SA and Smith GP (1989) Comparison of the effects of flavone acetic acid, fostriecin, homoharringtonine and tumour necrosis factor alpha on Colon 38 tumors in mice. *Eur J Cancer Clin Oncol* **25**: 263–269

Bendtzen K, Morling N, Fomsgaard A, Svenson M, Jacobsen B, Odum N and Svejgaard A (1988) Association between HLA-DR2 and production of tumour necrosis factor alpha and interleukin 1 by mononuclear cells activated by lipopolysaccharide. *Scand J Immunol* **28**: 599–606

Bruin KF (1994) *Endotoxin responsiveness in humans*. PhD Dissertation. University of Amsterdam, The Netherlands

Carswell EA, Old LJ, Kassel RL, Green S, Fiore N and Williamson B (1975) An endotoxin-induced serum factor that causes necrosis of tumors. *Proc Natl Acad Sci USA* **25**: 3666–3670

Ching L-M and Baguley BC (1987) Induction of natural killer cell activity by the antitumour compound flavone acetic acid (NSC 347512). *Eur J Cancer Clin Oncol* **23**: 1047–1050

Ching LM, Joseph WR and Baguley BC (1993) Inhibition of antitumor effects of flavone acetic acid by cortisone. *Anticancer Res* **13**: 1139–1141

Ching LM, Joseph WR, Crosier KE and Baguley BC (1994) Induction of tumor necrosis-factor-alpha messenger RNA in human and murine cells by the flavone acetic acid analogue 5,6-dimethylxanthenone-4-acetic acid (NSC 640488). *Cancer Res* **54**: 870–872

Chomczynski P and Sacchi N (1987) Single-step method of RNA isolation by acid guanidinium thiocyanate–phenol–chloroform extraction. *Anal Biochem* **162**: 156–159

Drouet C, Shakhov AN and Jongeneel CV (1991) Enhancers and transcription factors controlling the inducibility of the tumor necrosis factor-alpha promoter in primary macrophages. *J Immunol* **147**: 1694–1700

Finlay GJ, Smith GP, Fray LM and Baguley BC (1988) Effect of flavone acetic acid (NSC 347512) on Lewis lung carcinoma: evidence for an indirect effect. *J Natl Cancer Inst* **80**: 241–245

Futami H, Eader LA, Komschlies KL, Bull R, Gruys ME, Ortaldo JR, Young HL and Wiltrout RH (1991) Flavone acetic acid directly induces expression of cytokine genes in mouse splenic leukocytes but not in human peripheral blood leukocytes. *Cancer Res* **51**: 6596–6602

Haziot A, Tsuberi BZ and Goyert SM (1993) Neutrophil CD14 – biochemical properties and role in the secretion of tumor necrosis factor-alpha in response to lipopolysaccharide. *J Immunol* **150**: 5556–5565

Jacob CO, Fronek Z, Lewis GD, Koo M, Hansen JA and McDevitt HO (1990) Heritable major histocompatibility complex class II-associated differences in production of tumor necrosis factor alpha: relevance to genetic predisposition to systemic lupus erythematosus. *Proc Natl Acad Sci USA* **87**: 1233–1237

Kerr DJ and Kaye SB (1989) Flavone acetic acid – preclinical and clinical activity. *Eur J Cancer Clin Oncol* **25**: 1271–1272

Lee JD, Kato K, Tobias PS, Kirkland TN and Ulevitch RJ (1992) Transfection of CD14 into 70Z/3 cells dramatically enhances the sensitivity to complexes of lipopolysaccharide (LPS) and LPS binding protein. *J Exp Med* **175**: 1697–1705

Mahadevan V, Malik STA, Meager A, Fiers W, Lewis GP and Hart IR (1990) Role of tumor necrosis factor in flavone acetic acid-induced tumor vasculature shutdown. *Cancer Res* **50**: 5537–5542

Manus RM, Wilson AG, Mansfield J, Weir DG, Duff GW and Kelleher D (1996) TNF2, a polymorphism of the tumour necrosis-alpha gene promoter, is a component of the celiac disease major histocompatibility complex haplotype. *Eur J Immunol* **26**: 2113–2118

McKeage MJ, Kestell P, Denny WA and Baguley BC (1991). Plasma pharmacokinetics of the antitumour agents 5,6-dimethylxanthenone-4-acetic acid, xanthenone-4-acetic acid and flavone-8-acetic acid in mice. *Cancer Chemother Pharmacol* **28**: 409–413

Molvig J, Baek L, Christensen P, Manogue KR, Vlassara H, Platz P, Nielson LS, Svejgaard A and Nerup J (1988) Endotoxin-stimulated human monocyte secretion of interleukin 1, tumour necrosis factor alpha, and prostaglandin E2 shows stable interindividual differences. *Scand J Immunol* **27**: 705–716

O'Dwyer PJ, Shoemaker D, Zaharko DS, Grieshaber C, Plowman J, Corbett T, Valeriote F, King SA, Cradock J, Hoth DF and Leyland-Jones B (1987) Flavone acetic acid (LM 975, NSC 347512), a novel antitumor agent. *Cancer Chemother Pharmacol* **19**: 6–10

Perera PY, Barber SA, Ching LM and Vogel SN (1994) Activation of LPS-inducible genes by the antitumor agent 5,6-dimethylxanthenone-4-acetic acid in primary murine macrophages – dissection of signaling pathways leading to gene induction and tyrosine phosphorylation. *J Immunol* **153**: 4684–4693

Philpott M, Baguley BC and Ching L-M (1995) Induction of tumour necrosis factor-alpha by single and repeated doses of the antitumour agent 5,6-dimethylxanthenone-4-acetic acid. *Cancer Chemother Pharmacol* **36**: 143–148

Plowman J, Narayanan VL, Dykes D, Szarvasi E, Briet P, Yoder C and Paull KD (1986) Flavone acetic acid: a novel agent with preclinical antitumor activity against colon adenocarcinoma 38 in mice. *Cancer Treat Rep* **70**: 631–638

Pratesi G, Rodolfo M, Rovetta G and Parmiani G (1990) Role of T cells and tumour necrosis factor in antitumour activity and toxicity of flavone acetic acid. *Eur J Cancer* **26**: 1079–1083

Rewcastle GW, Kestell P, Baguley BC and Denny WA (1990) Light-induced breakdown of flavone acetic acid and xanthenone analogues in solution. *J Natl Cancer Inst* **82**: 528–529

Rewcastle GW, Atwell GJ, Zhuang L, Baguley BC and Denny WA (1991) Potential antitumor agents. 61. Structure–activity relationships for *in vivo* colon-38 activity among disubstituted 9-oxo-9H-xanthene-4-acetic acids. *J Med Chem* **34**: 217–222

Spies T, Morton CC, Nedospasov SA, Fiers W Pious D and Strominger JL (1986) Genes for the tumor necrosis factors alpha and beta are linked to the human major histocompatibility complex. *Proc Natl Acad Sci USA* **83**: 8699–8702

© Cancer Research Campaign 1997